# Egypt's Ambitious Strategy to Eliminate Hepatitis C Virus: A Case Study

**DOI:** 10.9745/GHSP-D-20-00234

**Published:** 2021-03-31

**Authors:** Ahmed Hassanin, Serageldin Kamel, Imam Waked, Meredith Fort

**Affiliations:** a Westchester Medical Center/New York Medical College, NY USA.; b Yale School of Medicine, New Haven, CT, USA.; c National Liver Institute, Shebeen El Kom, Egypt.; d Colorado School of Public Health, Aurora, CO, USA.

## Abstract

**Introduction:**

Chronic hepatitis C virus (HCV) infection is a major public health problem in many low- and middle-income countries. In 2015, Egypt's HCV infection prevalence of 7% among adults was among the highest in the world and accounted for 7.6% of the country's mortality. In 2014, Egypt embarked on an aggressive screening and treatment program that evolved into a national strategy to eliminate HCV as a public health threat by 2021.

**Methods:**

In this qualitative case study, we analyzed Egypt's HCV control strategy using the Kingdon framework to understand how the problem, policy, and political streams merged to create an opportunity to achieve an ambitious elimination goal. We describe key aspects of the implementation, identify lessons learned, and provide recommendations for other low- and middle-income countries aiming to eliminate HCV.

**Results:**

Between 2014 and 2020, Egypt screened more than 50 million and treated more than 4 million residents for HCV. Five key elements contributed to Egypt's successful HCV elimination program: (1) sufficient and reliable epidemiologic data to quantify and monitor public health threats; (2) a robust public health care infrastructure; (3) inclusive care that reached all sectors of society; (4) political commitment to public health through increased health care spending and a comprehensive long-term national control strategy; and (5) innovative scientific research and use of information technology.

**Conclusion:**

Egypt conducted a successful HCV screening program that covered more than 50 million residents and treated more than 4 million. It is poised to be the first country in the world to eliminate HCV within its borders. The lessons learned from this experience can inform the elimination plans of other low- and middle-income countries with high HCV burden.

## Abstract (precis)

A national hepatitis C virus elimination strategy rooted in mass screening and treatment can be effective in many middle-income countries. A strong public health infrastructure, political commitment, and technological advances are essential to such initiatives.

## Key Messages

- Despite the modest success of Egypt's first national control program for chronic hepatitis C virus in 2008, subsequent control and elimination campaigns between 2014 and 2020 successfully screened more than 50 million individuals and treated 4 million people for chronic hepatitis C.
- The campaign's success was driven by: (1) a robust public health care infrastructure and surveillance system; (2) political commitment to sustainable chronic hepatitis C virus elimination (3) inclusive care that reached all sectors of society; and (4) use of information technology and innovative scientific research.

## INTRODUCTION

### Context and Goals of Egypt's Hepatitis C Virus Elimination Program

Chronic hepatitis C is a liver infection that is caused by the hepatitis C virus (HCV). In 2015, an estimated 71 million people globally were living with HCV, 1.75 million people were newly infected, and the epidemic was responsible for an estimated 399,000 deaths.^1^ HCV-related deaths are primarily due to chronic (or end-stage) liver disease and liver cancer. Low- and middle-income countries accounted for about 75% of people living with HCV globally in 2016.^1^

Egypt, a lower middle-income country with a population of 100 million, had one of the highest burdens of HCV infections globally.^2^ In 2008, 15% of the population had antibodies to HCV (seropositive), indicating they had been exposed to the virus, and 1 in 10 Egyptians aged 15–59 years had chronic HCV infection.^3^ Faced with this major health and economic burden, Egypt established its first national control program for HCV in 2008, focused on expanding access to treatment. In 2014, Egypt issued its second national program for mitigating HCV, with emphasis on prevention, education, and improved patient care for those living with HCV.

**Figure:**
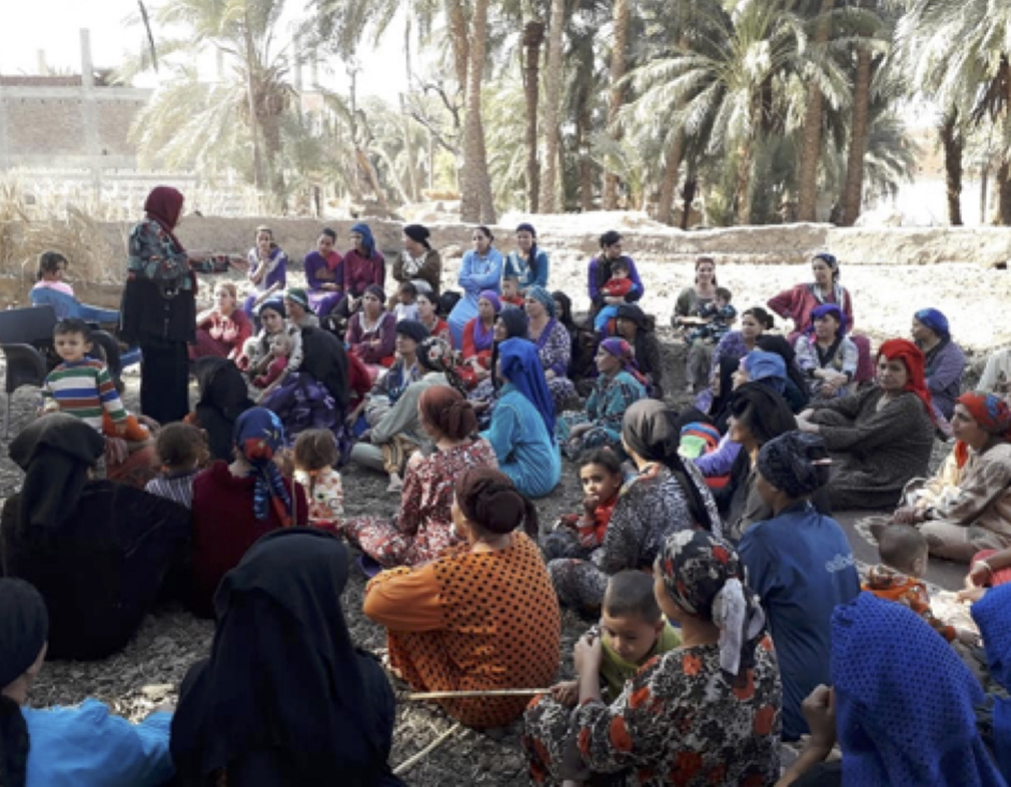
A community health worker educates a group of women about hepatitis C virus in a village in Assiut. © 2018 Tahsin Bakr

By 2018, the program had evolved into a national strategy to eliminate HCV as a public health threat. This new strategy aligned with the first Global Health Sector Strategy on Viral Hepatitis 2016–2021 agenda that was unanimously adopted by the 194 World Health Organization (WHO) member states, including Egypt.^4^ WHO signatories committed to eliminating viral hepatitis as a public health threat by 2030. Elimination was defined by WHO as a 65% reduction in mortality and a 90% reduction in incidence, compared with the 2015 baseline.^4^

### Purpose Statement

This case study examined Egypt's HCV control strategy from 2006 to the present, particularly 2014–2020. We aimed to describe Egypt's HCV control strategy using the Kingdon framework^5^ to understand how the problem, policy, and political streams merged to create the window of opportunity for a successful HCV elimination program. We examined the factors and challenges associated with the HCV elimination program's creation, implementation, and results. We then summarized the lessons learned from the program. These lessons can inform HCV elimination plans in other low- and middle-income countries.

We conducted an extensive review of documents published from 2006 to 2020 and supplemented this review by consulting key stakeholders. Data sources included World Bank reports; World Health Organization documents; official Egyptian governmental publications; National Committee for Control of Viral Hepatitis (NCCVH) strategy and action plans; published statements by key stakeholders involved with the program, including the Ministry of Health and Population (MOHP), NCCVH members, WHO director-general, and frontline workers; and peer-reviewed publications (Box 1). To identify peer-reviewed publications, we conducted a PubMed search using the search terms “Egypt” and “Hepatitis C.” We used an iterative process to review official and peer-reviewed publications, in which newly collected data were extracted, discussed, and categorized by the coauthors. We discussed the pertinence of specific events and data points as they related to the 3 streams of the Kingdon framework and to the lessons learned from the Egyptian experience until consensus was reached.

Box 1 **Key Stakeholders Involved in Egypt's Hepatitis C Virus Elimination Program, 2016–2021**

- National Committee for Control of Viral Hepatitis (NCCVH): Established in 2006, the NCCVH's goals include monitoring hepatitis C virus (HCV) incidence and prevalence, developing a strategy to combat the spread of HCV, and establishing a wide network of specialized centers to provide integrated care for HCV patients. The NCCVH comprises hepatology and public health experts from Egypt and abroad and is divided into an advisory board and an executive working group.
- Egyptian government and political leadership: The Ministry of Health and Population played a major role in rolling out HCV prevention and treatment strategies, as well as establishing training and education activities for thousands of health care personnel.
- Egyptian public: HCV has been a mainstream health concern for many Egyptians since the late 1900s. A serologic survey conducted as part of the 2008 Demographic and Health Survey showed that 1 in 10 Egyptian adults aged 15–59 years had chronic HCV infection.^3^
- Nongovernmental organizations (NGOs) and media: Several NGOs contributed to HCV screening efforts, community-based education, and supporting the treatment costs for uninsured patients. The local media played a major role in raising awareness about HCV and promoting screening and treatment efforts.
- Foreign organizations: The U.S. Centers for Disease Control and Prevention, the U.S. Naval Medical Research Unit 3 based in Cairo, and the Pasteur Institute of France provided technical support in drafting the national HCV control strategies.
- World Health Organization: The Global Health Sector Strategy on Viral Hepatitis 2016–2021 document^4^ endorsed by the World Health Assembly served as a blueprint for Egypt's HCV elimination strategy starting in 2017.
- World Bank: The World Bank played 2 key roles in support of Egypt's HCV control program: (1) assisting with feasibility studies of various HCV elimination programs^6^^,^^7^; and (2) providing monetary assistance in the form of loans to the health care sector.^8^
- Pharmaceutical and medical device companies: The Egyptian government worked closely with several local and international companies to ensure adequate supply of HCV testing supplies and therapeutic drugs.

**FIGURE 1:**
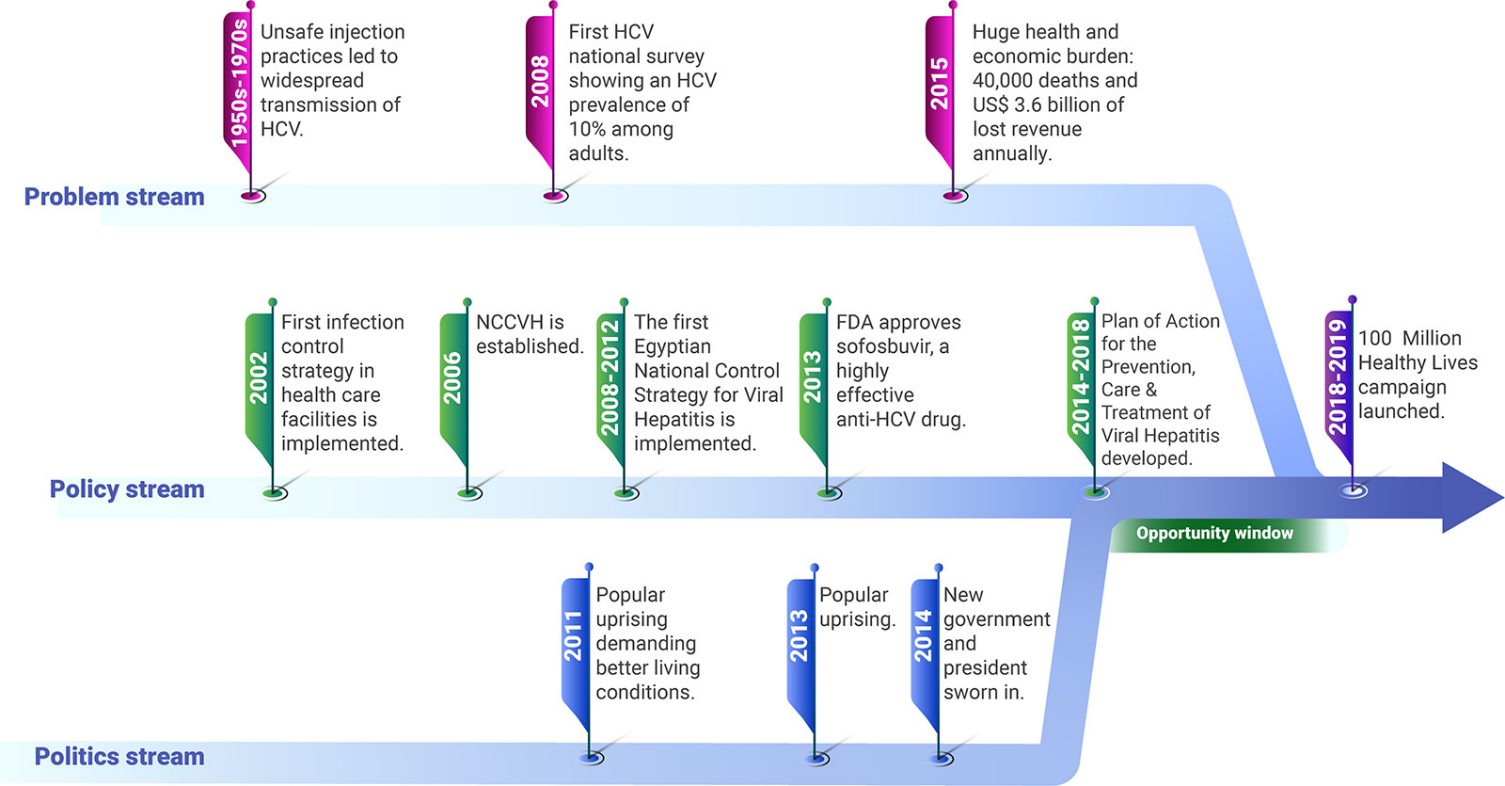
Timeline of the 3 Streams of the Kingdon Framework That Led to Defining Elimination of Hepatitis C Virus Goal Abbreviations: FDA, U.S. Food and Drug Administration; HCV, hepatitis C virus; NCCVH, National Committee for Control of Viral Hepatitis.

Figure 1 presents a summary of the key moments in each of the 3 streams that led to defining elimination as a goal in 2018 and 2019. The following sections describe these streams in detail.

### Problem Stream

The origins of the HCV epidemic in Egypt can be traced back to the 1950s–1980s, during a mass treatment campaign for the parasitic illness schistosomiasis, or bilharzia. The use of inadequately sterilized, reusable needles during this campaign led to widespread transmission of HCV.^9^^,^^10^ Over the last 2 decades, the majority of new HCV cases in Egypt were attributed to HCV transmission in health care facilities related to blood transfusions and suboptimal infection control techniques.^11^^–^^13^ Until 2007, no large nationwide HCV prevalence studies had been conducted. Government insurance schemes did not cover the cost of HCV therapy, and infection control policies were limited. In a 2012 presentation, NCCVH board member Dr. Gamal Esmat stated^14^:

*Unfortunately, till 2007, we did not have a national control program for control of viral hepatitis.*

The 2008 Demographic and Health Survey revealed that 15% of Egypt's adult population had antibodies to HCV (seropositive), indicating they had been infected with the virus, and 1 in 10 Egyptians aged 15–59 years had chronic HCV infection.^3^ A second survey in 2015 estimated that 7% of Egypt's population aged 19–65, roughly 4.1 million people, had chronic HCV infection.^15^ This number included 750,000 previously diagnosed individuals who were on a waitlist pending access to treatment.^16^ That year, the burden of HCV accounted for 40,000 deaths,^7^ or 7.6% of all deaths, and a loss in life expectancy of approximately 1.8 years for men and 1.0 year for women. The World Bank estimated the total economic burden of HCV in Egypt to be US$3.81 billion, equivalent to 1.4% of the country's total GDP, in 2015.^7^^,^^17^

Medical and public health experts recognized the HCV epidemic in Egypt as of the early 2000s. Yet, several studies suggest a poor understanding of HCV modes of transmission and therapeutic options among Egyptians.^18^^,^^19^ By the end of 2011, only 2.8% of patients with HCV had started treatment, and only 1.67% of the total number achieved sustained virologic response (cure).^9^ Early on, the NCCVH faced several challenges, including high costs, extensive adverse effects, and low cure rates of therapies available at the time.

### Policy Stream

Although Egypt's response to HCV as a public health crisis intensified significantly in 2014, much of the experience and physical infrastructure of the HCV control program had been established nearly a decade earlier. The NCCVH was established in 2006 to combat the spread of HCV and establish the National Network of Treatment Centers (NNTC) to provide specialized, integrated care for HCV patients. In 2008, the NCCVH published the first Egyptian National Control Strategy for Viral Hepatitis.^14^ The strategy comprised several main goals for 2008–2012:

- Detect the prevalence and incidence of HCV and hepatitis B virus (HBV) infections.
- Reduce the prevalence of chronic HCV and HBV infections in the 15–30 age group by 20% of 2008 levels by 2012.
- Expand access to treatment to within 100 km for all Egyptians and treat 50% of individuals needing treatment by 2012.
- Continue to produce high-quality scientific research.
- Ensure programmatic sustainability.

The plan achieved several of its goals:

- The government acknowledged the magnitude of the HCV problem in Egypt, as supported by publishing the 2008 Demographic and Health Survey, which showed an HCV prevalence of 10% among Egyptians aged 15 to 59 years.
- National guidelines for the treatment of chronic HCV were established.
- Between 2007 and 2010, 21 specialized HCV treatment centers were established.
- Governmental funding for HCV treatment programs exceeded 90%.

The strategic plan was a modest success—only about 191,000 Egyptians had started treatment.^20^ At the time, treatment modalities included a combination of pegylated interferon and ribavirin given over an extended period. These treatments were expensive, with extensive adverse effects and a low cure rate of about 60%. The underlying investment of US$80 million in subsidized HCV treatment over 4 years was inadequate.^21^ From 2008 to 2012, the MOHP emphasized HCV treatment more than prevention of transmission among high-risk groups.^22^

In December 2013, the panorama of HCV treatment changed dramatically when the U.S. Food and Drug Administration approved sofosbuvir, a new class of HCV direct-acting antiviral drug. Sofosbuvir in combination with other antivirals offered up to a 90–95% sustained virologic response, or cure rate, using a simple and well-tolerated 12-week oral regimen.^23^ However, the retail price of this treatment was US$84,000 per individual case. Gilead, the pharmaceutical company that owned the patent for sofosbuvir, applied for a proprietary license in Egypt. The application was initially rejected, forcing the company to enter negotiations with the Egyptian government. These negotiations resulted in a voluntary licensing agreement in March of 2014. Sofosbuvir would be sold to the Egyptian government at a 99% discount, or US$900 per treatment course.^24^ Eventually, sofosbuvir was licensed to several Egyptian pharmaceutical companies. Between 2015 and 2018, it cost just under US$84 for the typical 12-week treatment course.^25^

Egypt's second HCV national strategy, covering 2014–2018, is referred to as the Plan of Action for the Prevention, Care & Treatment of Viral Hepatitis.^26^ The plan aimed to build on the success of clinical trials for direct-acting antiviral drugs, to overcome the limitations of the previous strategy by emphasizing reduced HCV transmission via increased prevention and education, and to ensure access to safe and effective care and treatment for all Egyptians. The 2 main goals of this plan were to: (1) prevent HCV transmission and treat HCV patients on the treatment waitlist, and (2) offer treatment to 300,000 patients annually. These goals were implemented along 5 predefined axes (Table).^27^^–^^33^

**Figure:**
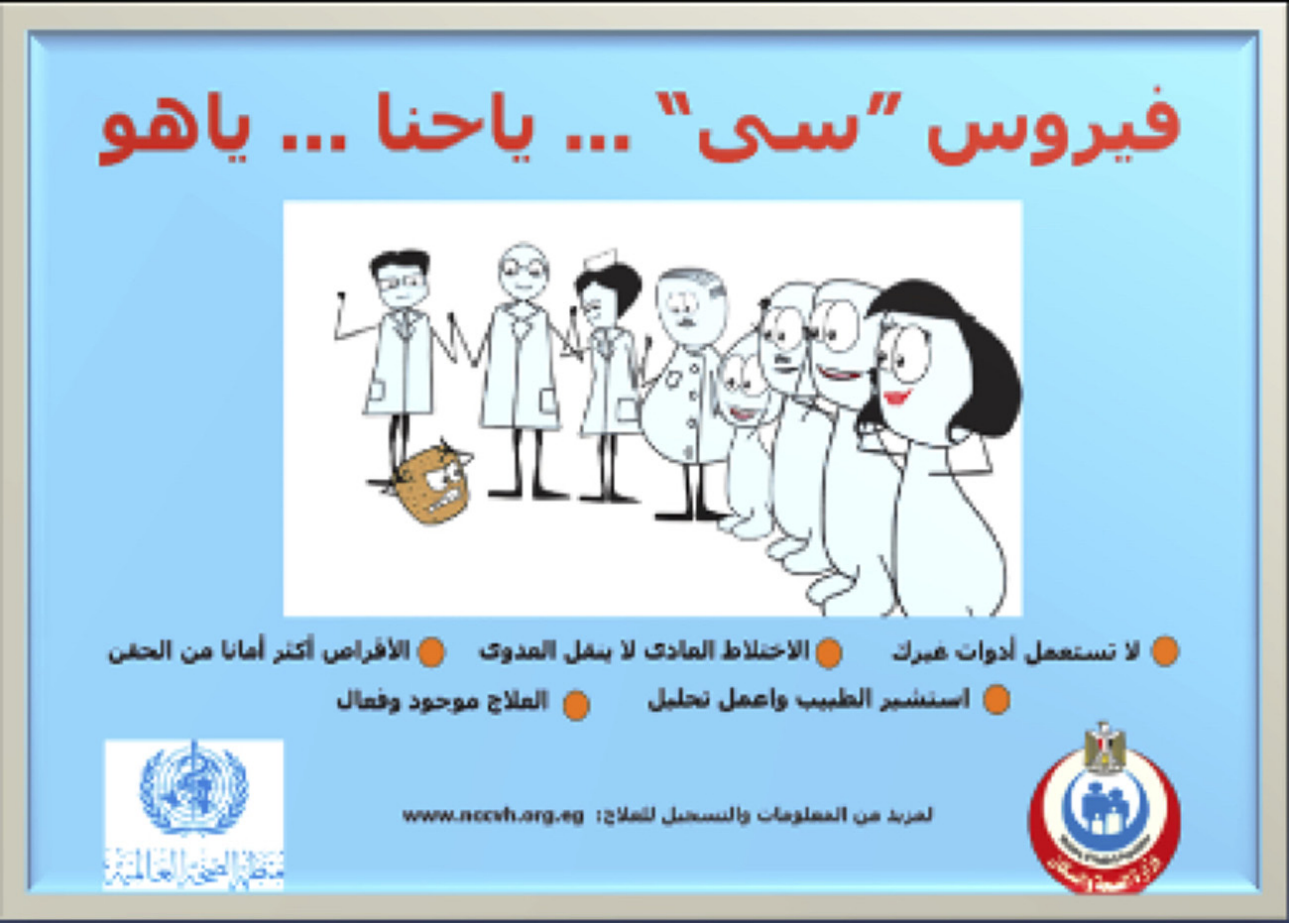
Advertisements in Arabic used in communication strategy for hepatitis C virus that conveyed 5 messages. “It is either us, or hepatitis virus C: (1) Don't use personal items that are not yours; (2) It is ok to have contact with HCV patients; (3) Pills are safer than injections; (4) Consult with a physicians and test for HCV; and (5) HCV treatment is available and effective.” © World Health Organization Regional Office for the Eastern Mediterranean

**TABLE.** Five Axes for Implementing Egypt's Viral Hepatitis Plan of Action, 2014–2018

Axis	Actions
Axis 1: Strengthening surveillance to detect viral hepatitis transmission and disease	The Demographic and Health Survey conducted in 2015 updated the HCV prevalence estimate in adults aged 15–59 years from 10% to 7%, or approximately 4.1 million Egyptians. The survey provided a detailed breakdown of HCV prevalence in rural versus urban areas, between various administrative governorates, and across different socioeconomic strata.
Axis 2: Improving blood products safety to reduce transmission of viral hepatitis	Transmission of HCV via blood product transfusion is among the most common modes of HCV infection in Egypt. Policies to prevent such transmission were implemented, and nucleic-acid testing for all blood products in Egypt became mandatory in private and public health care facilities. Screening for HCV for all pregnant women also became mandatory.
Axis 3: Promoting infection control practices to reduce transmission of viral hepatitis	MOHP, WHO, CDC, and the biomedical research lab of the U.S. Navy located in Cairo, Egypt, updated national infection control policies and guidelines and launched a national training and auditing campaign with health care workers and facilities to ensure adherence to infection control policies.^25^ This comprehensive plan launched in 2002 and included policies for prevention of transmission in health care facilities and dialysis centers. It was relaunched in 2014 as part of the Plan of Action for the Prevention, Care & Treatment of Viral Hepatitis. It had a significant impact on hospital-acquired HCV transmission. Among dialysis patients, the annual HCV incidence rate declined from 28% in 2001 to 6% in 2012^26^ and just 1.6% in 2015.^27^
Axis 4: Educating providers and communities to increase awareness about viral hepatitis and its prevention	Nongovernmental organizations contributed to community-based education and test-and-treat projects seeking to raise awareness and promote behavioral changes that reduce HCV transmission.^28^ WHO provided technical support to the MOHP in drafting a 5-year communication strategy and implementation plan to combat HCV.^29^ The communication strategy focused on 5 main messages, and 5 television outlets, 5 radio outlets, and online social media were used to spread the message. A library of the visual advertisements and messaging is available on the MOHP-sponsored YouTube channel.^30^
Axis 5: Improving care and treatment to prevent liver disease and cancer	In September 2014, the NCCVH debuted an online portal for people living with HCV to register for treatment. Using this portal, people could register for treatment by inputting basic demographic data, their national identification number, and a call-back number. Within 24 hours, an operator from the call center would call to set up an appointment and give an overview of expectations for the upcoming visit. In the first month after opening the online portal, over 500,000 people registered to receive HCV treatment. To manage this influx of patients, the number of comprehensive treatment centers across the 27 administrative governorates was increased from 21 centers in 2010 to 54 centers in 2014, with less than 50 km between any two centers. By 2018, the number of centers expanded to 121.^31^ The treatment centers, which formed the NNTC for HCV, were integrated care units that provide all required services for HCV management. A well-trained, multidisciplinary team of medical doctors, pharmacists, nurses, and administrative staff participated in the assessment and follow-up of each patient. By September 2018, 2.4 million people with HCV had been treated using various sofosbuvir-based direct-acting antiviral drug combinations for 12 or 24 weeks, depending on the presence or absence of cirrhosis (liver failure).^22^^,^^32^ The number of patients treated reflected the number of previously recognized cases awaiting access to treatment and newly diagnosed cases due to expanded screening.

Abbreviations: CDC, U.S. Centers for Disease Control and Prevention; HCV, hepatitis C virus, MOHP, Ministry of Health and Population; NCCVH, National Committee for Control of Viral Hepatitis; NNTC, National Network of Treatment Centers; WHO, World Health Organization.

### Political Stream

Egypt experienced popular uprisings in 2011 and 2013, leading to changes in the political regimen, including a new government and president in 2014. The prospect of significantly reducing the burden of HCV in Egypt was seen by the new administration as a means to galvanize the country around a common goal of improving public health. At the time, elimination of HCV seemed far-fetched, so the administration's emphasis was on offering treatment to previously confirmed HCV patients who were still awaiting treatment and limiting HCV transmission.

In 2016, the 194 WHO member states, including Egypt, committed to eliminating viral hepatitis as a public health threat by 2030. The same year, the Egyptian government consulted with the World Bank to model several scenarios. One such scenario—the “current path”—screened 5% of the population per year in addition to continuing demand-driven treatment. An HCV elimination scenario screened 20% of at-risk individuals per year, positive cases would be offered treatment, and the entire population would be covered by 2022.^6^^,^^7^ The elimination scenario would cost US$530 million more in the short term, but it eventually would reduce the health budget by an estimated $60 million every year between 2023 and 2030 by preventing HCV-related complications. Building on the success of the 2014–2018 HCV national strategy the government shifted its strategy from HCV control to elimination.

## THE 3 STREAMS MERGE: 100 MILLION HEALTHY LIVES CAMPAIGN

In late 2018, the government launched the 100 Million Seha (100 Million Healthy Lives) campaign under the patronage of Egyptian President Abdel-Fattah Elsisi. The campaign aimed to eliminate HCV as a public health threat.^34^ It included free voluntary HCV screening to all residents of Egypt aged 18 years and older, or about 62 million people, and offered free treatment for confirmed cases. Between October 2018 and April 2019, more than 60,000 health care personnel (e.g., doctors, nurses, pharmacists, lab technicians, and data entry employees) participated in the campaign at more than 5,000 HCV screening or treatment sites.^35^

### Implementation

#### Funding Sources

The Egyptian government has substantially increased health care spending over the past decade. Between 2010 and 2017, governmental health care spending increased from 4.3% to 5.2% of total government spending, a per-capita increase from US$141 to US$205.^36^ The main funds for the HCV elimination program involved governmental funding for the MOHP to support screening and treatment of uninsured individuals. The Transforming Egypt's Healthcare System Project, a 5-year project that commenced in 2018, is a US$992.5 million government plan targeting improvement in the quality of primary, secondary, and family planning health care services, as well as the prevention and treatment of HCV.^8^ From the total budget of the project, US$129.6 million was assigned to HCV screening, US$130.6 million to HCV treatment, and US$50 million to the National Blood Transfusion Services system to improve screening of blood products for transmissible infections. Later the same year, the World Bank approved a US$530 million loan to support the Transforming Egypt's Healthcare System Project.^8^

#### Monitoring and Quality Assurance Mechanisms

One of the accomplishments of the first Egyptian National Control Strategy for Viral Hepatitis between 2008 and 2012 was the establishment of a closed virtual private network to connect the NNTC to the NCCVH head office to collect real-time patient data from a unified case report. The virtual private network facilitated clinical and operational monitoring by NCCVH in real time so that case volume and treatment quality could be monitored at the regional and center levels.^37^ Additionally, it allowed for instant troubleshooting to overcome logistical challenges. The integration of health information systems reduced paperwork costs, which accounted for 65% of patients' out-of-pocket costs.^35^

The NCCVH treatment regimens have changed over time to reflect the most recent data, availability, and cost-effectiveness of various treatment regimens. A study covering all HCV patients in Egypt treated between October 2014 and March 2016 compared and contrasted the treatment regimens of more than 330,000 patients to identify the most effective ones.^23^ The data aided in choosing the most effective regimens for use when HCV elimination became a stated goal.

#### Coverage

**FIGURE 2:**
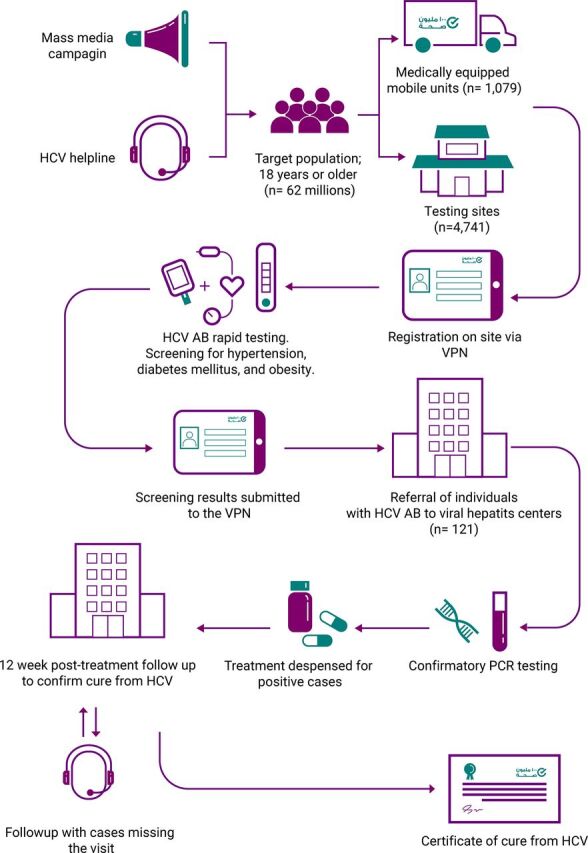
Flow of Individuals Screened for Hepatitis C Virus Through by the 100 Million Health Lives Campaign in Egypt Abbreviations: AB, antibody; HCV, hepatitis C virus; PCR, polymerase chain reaction; VPN, Virtual Private Network.

Figure 2 illustrates the flow of individuals reached by the 100 Million Health Lives Campaign. Patients diagnosed with but not treated for HCV registered at a website (stophcv.eg). Within 24 hours of registering, the person received a call from an operator to schedule an appointment at a nearby care center within the NNTC. Individuals with no known history of HCV also could be screened for HCV without an appointment at any of the 5,820 testing sites (including 1,079 mobile units) throughout the country.^35^ These individuals were tested onsite and typically received their results within 20 minutes. Individuals with positive screening tests were referred to the nearest NNTC member for confirmatory testing. Confirmed HCV cases were referred for treatment, which was typically approved within 1 week. All patients who were treated for HCV were asked to return to the treatment center after completing the 12-week therapy to evaluate their sustained virologic response (cure status) of HCV.

#### Adaptations

An important lesson learned between 2014 and 2016 was that prioritization based on the presence of advanced liver fibrosis (damage), which required patients to undergo a liver biopsy, caused significant backlogs and impaired enrollment in the treatment program.^23^ Initially, the limited supplies of medication, high costs, and capacities of treatment centers made prioritization necessary. As the availability of drugs and treatment centers increased, however, individuals with HCV at all stages of fibrosis, including no evidence of fibrosis, were treated. At that time, Egypt became the first country globally to treat all people with HCV, regardless of their fibrosis stage.

Egypt's HCV elimination efforts intensified with the inauguration of the 100 Million Healthy Lives campaign. Efficiency became a priority. To screen 60 million eligible Egyptians, the campaign was divided into 3 phases, each comprising 7 to 11 administrative governorates, at 5,820 testing sites throughout the country.^35^ To serve the 57% of Egyptians who live in rural areas,^38^ 1,079 medically equipped vehicles were used to reach remote and underserved areas.

**Figure:**
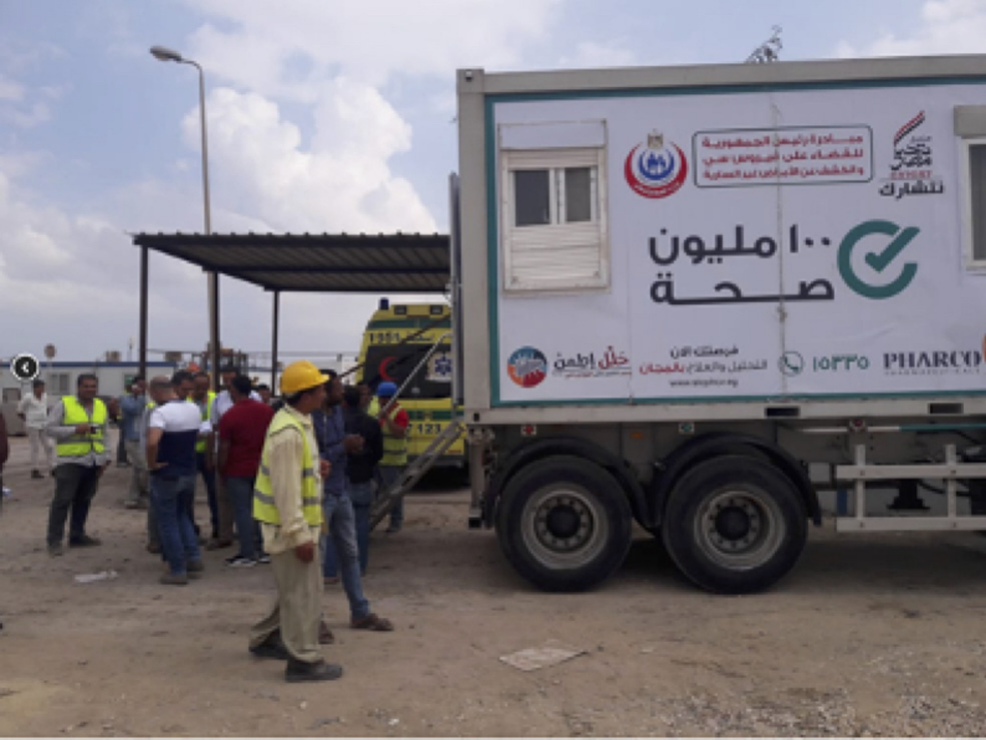
A medically equipped vehicle used for screening individuals for hepatitis C virus near a construction site in South Sinai, Egypt. © 2018 Tahsin Bakr

The overall rate of compliance with post-treatment follow-up visits increased from 28% in 2014 to 75.4% in 2016.^23^ Compliance reached 85% during the 100 Million Health Lives Campaign. Several methods were adopted to encourage return visits to evaluate patients' sustained virologic response. These methods included issuing a “certificate of cure” to those who had a negative viral load test at 12-weeks post-treatment. To encourage patients to return for evaluation of their sustained virologic response, the HBV vaccine was offered free of cost at the end of HCV treatment. Patients with HCV but no history of HBV were given the first dose of the HBV vaccine at their last HCV treatment appointment. They were asked to return 12 weeks later for the second HBV vaccine dose as part of HCV post-treatment follow up visit intended to confirm sustained virologic response. A professional call center was contracted to contact patients who did not show up for treatment appointments and treated patients who did not show for their 12-week post-treatment evaluation. The call center recorded the reason for their “no-show” and set new appointments if needed.

To encourage HCV screening among low-risk young adults who might be reluctant to get tested, the screening sites additionally offered screening for diabetes, hypertension, and obesity to attract individuals who desired a general health check. All screening data were electronically recorded. Individuals with abnormal blood pressure or blood glucose levels were referred to the nearest primary care unit.

#### Acceptability

The 100 Million Health Lives campaign was widely accepted by the public for several reasons:

- The high magnitude of effort was perceived by the public as a serious attempt to eliminate HCV as a public health threat.
- Conducting the program under the patronage of Egyptian President Abdel-Fattah Elsisi signaled that it was a national priority.
- Major political figures participated in the screening campaign, which had mass media coverage.
- Screening sites offered widespread availability, operating 12 hours per day 7 days per week, to overcome logistical challenges that many individuals who were interested in screening might face.
- The mass media campaign that accompanied the launch of the 100 Million Healthy Lives campaign focused on educating the public on HCV and reducing the stigma associated with HCV diagnosis.
- Screening and treatment for HCV, as well as follow-up visits, were free for participants.

### Program Evaluation

#### Results of the Campaign

When the 100 Million Health Lives campaign concluded, the Egyptian MOHP announced that nearly 50 million Egyptians and 36,000 foreign residents of the intended 62 million were screened for HCV.^35^ Of those, 2.2 million individuals were seropositive, indicating HCV exposure or chronic infection, and referred for confirmatory testing. Of those referrals, 1.6 million patients had confirmed chronic HCV infection. Many were unaware of their status, as noted by a 47-year-old patient from Alexandria who tested positive:

*I didn't know I was infected with hepatitis C. I had no symptoms.*

**FIGURE 3:**
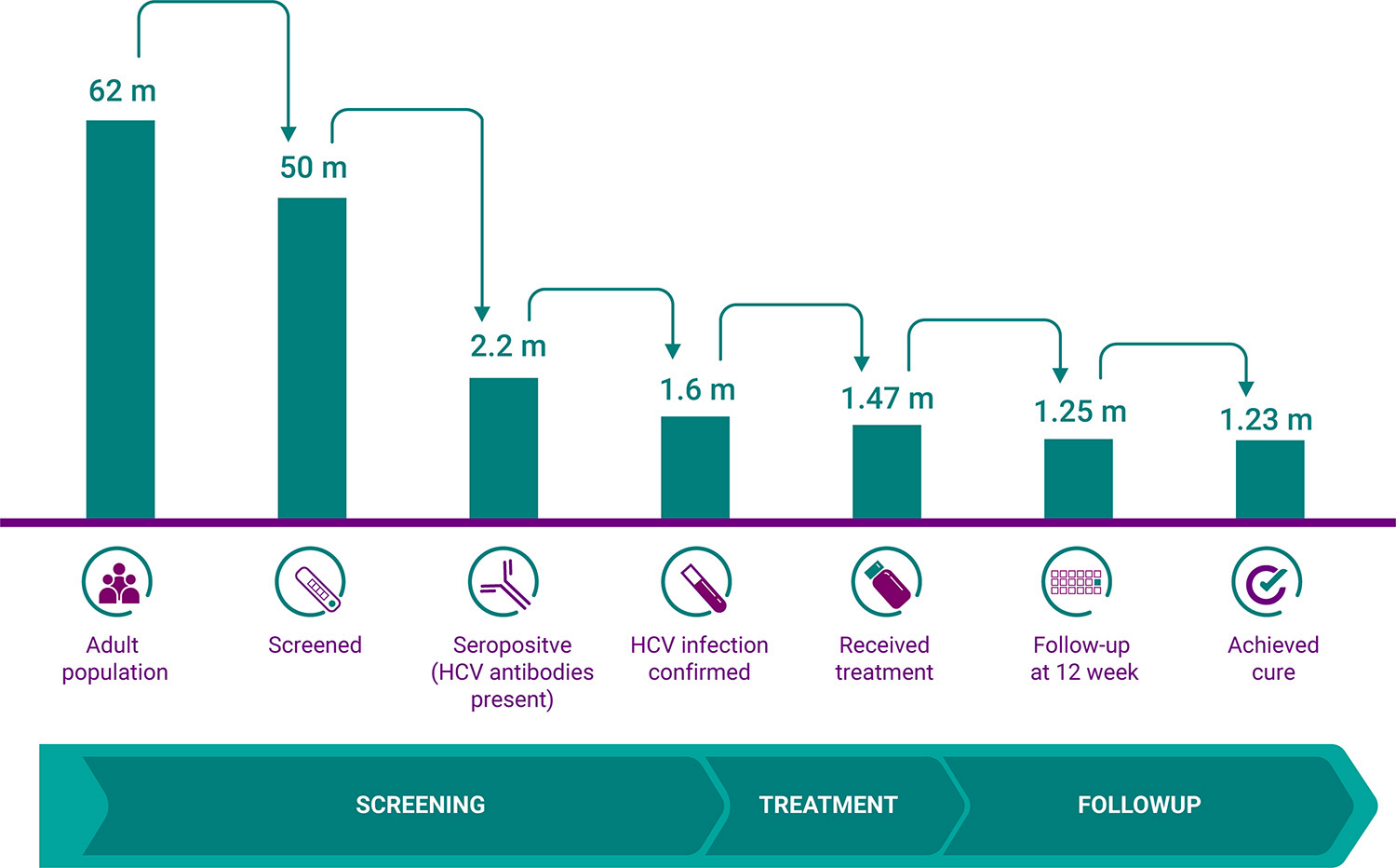
Hepatitis C Virus Screening and Case Cascade Used by Egypt's 100 Million Health Lives Campaign Abbreviation: HCV, hepatitis C virus.

Over the course of the 7-month campaign, 900,000 confirmed cases received treatment.^39^ An additional 700,000 confirmed cases were treated after the conclusion of the campaign. The rate of sustained virologic response (cure) was 98%.^23^ In February 2020, Dr. Hala Zayed of the Ministry of Public Health announced that between March 2014 and January 2020, 4 million Egyptians had been treated for HCV through the NNTC.^40^ Figure 3 shows the care cascade describing patient retention across sequential stages of the 100 Million Health Lives campaign and the subsequent continuum of care for diagnosed HCV cases.

After screening all willing adults for HCV, the 100 Million Healthy Lives campaign shifted its attention to HCV screening of students aged 6 to 18, after obtaining consent from their legal guardians. Khaled Megahed, spokesperson for the MOHP, stated that 3.8 million students across Egypt were screened between May 2019 and January 2020.^41^ In June 2020, Dr. Hala Zayed announced that Egypt had become the first country in the world to overcome hepatitis C^42^:

*Egypt proved that community-based screening of hepatitis viruses is not impossible.*

#### Costs

The Egyptian government procured a license to locally manufacture direct-acting antiviral drugs, reducing the price for a typical 12-week treatment course to US$84 between 2015–2018 and then further to US$54 in 2018. The government also purchased HCV screening kits (SD Bioline HCV Abbott) at costs as low as US$0.60 per kit and real-time quantitative polymerase-chain reaction assays (Cobas AmpliPrep/CobasTaqMan HCV Test, Roche Diagnostics) at US$4.80 per test.^43^ This decreased the total cost of identifying a patient with HCV viremia to US$85.41 and the cost of identifying and curing a patient to US$130.62.^43^ Testing and treatment were fully funded by the state, irrespective of one's financial ability or insurance coverage. Of all patients tested, 58% were covered by the NCCVH, 24% by the Egyptian Health Insurance Organization, and 15% paid out-of-pocket to secure brand name direct-acting antiviral drug combinations.^32^ The testing and treatment component of the 100 Million Healthy Lives campaign had a direct total cost of US$207.1 million.^43^ The total cost of the HCV elimination effort since 2014 has not been made public, but 2017 World Bank projections estimate the excess cost of HCV elimination incurred by the Egyptian health care sector at US$530 million.^7^

## SYNTHESIS

### Lessons Learned

The main lessons gleaned from Egypt's HCV control program over the past decade and a half can be viewed through the lens of WHO's Global Health Sector Strategy on Viral Hepatitis 2016–2021 to achieve the 2030 elimination target (Box 2). WHO Director-General Dr. Tedros Adhanom Ghebreyesus visited the command center for the 100 Million Healthy Lives campaign. In response to that visit, he tweeted in August 2019^44^:

*During my visit to #Egypt I saw three things that are key to success: speed, scale and quality. This is a great example to follow on the way to achieve #HealthForAll!*

- Speed: A central-level commitment to swift elimination of HCV helped make this achievement possible. Over 7 months, nearly 50 million Egyptians were screened. Between 2014 and 2020, more than 4 million Egyptians were treated for HCV. This rapidity helped galvanize governmental agencies and the public around a common goal with a target date.
- Scale: The Egyptian government capitalized on a strong public health infrastructure and a highly centralized command system to scale up the campaign on a national level, mobilizing all of its resources to that end. It also took advantage of mass purchasing to drive prices down.
- Quality: The NCCVH used an evidence-based, adaptable approach to HCV elimination built on locally and internationally produced scientific research. The MOHP used information software systems to ensure equity and efficacy of HCV screening and treatment across the population.

Box 2 **Key Lessons Gleaned From Egypt's Hepatitis C Virus Control Program, 2014–2020**

- Information for focused action: National disease surveys are key to understanding the magnitude and progression of public health problems. Use of information technology systems is essential for accurate monitoring and success of implemented care policies.
- Interventions for impact: A strong public health system infrastructure and steadfast political commitment are essential for providing a package of high-impact interventions based on local and international expertise.
- Delivering for equity: A commitment to reaching out to the sectors of society that are most likely to be overlooked and to adapting the approach to those sectors is essential to ensure quality.
- Financing for sustainability: Increasing governmental health care spending and the support of international organizations concerned with health are important steps for the sustainability of national-level healthcare programs. Mass purchasing can reduce costs. Upfront spending can translate to downstream savings in healthcare spending and economic revenue.
- Innovation for acceleration: Locally produced scientific research, evidence-based decisions, and program adaptability are essential in any large health care program.

The lessons learned in Egypt over the past decade and a half can be viewed through the lens of WHO's Global Health Sector Strategy on Viral Hepatitis 2016–2021 to achieve the 2030 elimination target (see Box 2).

The Egyptian experience with HCV elimination can serve as a model for other low- and middle-income countries with high HCV burdens. The Coalition for Global Hepatitis Elimination, launched in July 2019, provides services and resources to assist in the planning, implementation, and evaluation of national and sub-national programs to eliminate HCV transmission and disease.^45^

At the 2019 African Hepatitis Summit, Egyptian Minister of Health and Population Dr. Hala Zaid announced that Egypt would provide HCV testing and treatment for 1 million people in 14 African countries^46^:

*Egypt has pledged to provide technical support, expertise and screening software, as well as free treatment for 1 million of our African sisters and brothers with hepatitis C for 3 months as part of our role on the continent*.

In a June 2020 news report, the Egyptian MOHP announced that since the inception of the program, 30,632 African citizens from South Sudan, Chad, and Eritrea had been tested for hepatitis C, and 376 citizens had received free treatment.^47^

### Program Weaknesses

Some challenges to the success of the elimination effort included low post-treatment follow-up testing rates and people not getting screened. Nearly 12.5 million Egyptian adults (20% of eligible individuals) were not screened. Although the reason for this non-participation is unknown, a potential explanation is that 10 million Egyptians live and work abroad. Additionally, although infection control measures in health care settings have significantly improved over the past decade, several areas in the domain of primary prevention have not been adequately addressed, such as limiting HCV transmission via IV drug use, sex workers, and barbershops.

### Sustainability

The NCCVH plans to sustain HCV-related gains by instituting the following strategies (email communication with NCCVH board, December 11, 2021):

- Screening of all pregnant women for HCV and HBV
- HCV screening of all first graders in preparatory school and students commencing university education over the next 5 years
- HCV and HBV rescreening and treatment for at-risk individuals who missed the national screening program, including persons who inject drugs, who are incarcerated, and who are on dialysis
- Establishment of a harm-reduction program for persons who inject drugs, including syringe distribution
- Establishment of a national follow-up program for the nearly 400,000 people who have cirrhosis or history of cirrhosis to promote early detection of liver cancer and decompensation, as HCV among these individuals can be more difficult to cure.

## CONCLUSION

Between 2014 and 2020, Egypt screened more than 50 million and treated 4 million residents for chronic HCV, with the goal of eliminating HCV as a public health threat. Five key elements led to the success of this elimination program: (1) a reliable epidemiologic surveillance to quantify and monitor public health threats; (2) a robust public health care infrastructure; (3) inclusive care that reached all sectors of society; (4) political commitment to public health through increased health care spending and a comprehensive long-term national control strategy; and (5) innovative scientific research and use of information technology. Egypt is poised to be the first country in the world to eliminate HCV within its borders. The lessons learned from this experience will inform the elimination plan of other low- and middle-income countries with high HCV burden.

## Notes

**Peer Reviewed**
